# Truncation of retinoschisin protein associated with a novel splice site mutation in the RS1 gene

**Published:** 2008-08-25

**Authors:** Balázs Lesch, Viktória Szabó, Melinda Kánya, Balázs Varsányi, Gábor M. Somfai, János Hargitai, Rita Vámos, Orsolya Fiedler, Ágnes Farkas

**Affiliations:** 1 Department of Ophthalmology, Semmelweis University, Budapest, Hungary; 2 First Department of Pathology and Experimental Cancer Research, Semmelweis University, Budapest, Hungary; 3 OBIK Medical Biotechnology Innovation Center Inc., Budapest, Hungary

## Abstract

**Purpose:**

To present the ocular findings of a Hungarian family with X-linked juvenile retinoschisis (XLRS) and to reveal a novel putative splice mutation leading to serious truncation of retinoschisin (RS1) protein. Our genetic results were compared to a mouse model of XLRS.

**Methods:**

Complete ophthalmic examinations were performed on five members (two male patients, two female carriers, and one healthy fraternal male twin) of the family. The examinations included optical coherence tomography (OCT) and full-field and multifocal electroretinography (mfERG). OCT and ERG results were compared to the normative database of our laboratory. All exons and the flanking intronic regions of the *RS1* gene were amplified by polymerase chain reaction and directly sequenced in all family members and in 50 male controls.

**Results:**

Typical microcystic foveal changes were found on fundoscopy and OCT in two male patients. Large foveal and smaller perifoveal cysts were detected by OCT in the inner nuclear layer and another deeper retinal cleavage in the photoreceptor layer. The standard combined b-wave amplitudes and b/a amplitude ratios of full-field ERGs of the male patients were decreased compared with controls, but the typical “negative-type” ERG was not observed. The amplitudes of mfERGs were reduced in all rings but mainly in the central part of the examined retina. Implicit times were delayed across almost the whole testing field. Female carriers and the healthy fraternal twin brother were without any symptoms and had normal clinical examination results, but the implicit times of female carriers were delayed in all rings. DNA sequence analyses revealed a novel putative splice mutation (c.78+1G>C) in the splice donor site of intron 2 in *RS1* of two male patients and two female carriers. Mutations were absent in the 50 control samples.

**Conclusions:**

Male patients exhibited typical bilateral foveal retinoschisis in two retinal layers and characteristic ERG changes. The inheritance of the novel putative splice mutation (c.78+1G>C) followed the classic inheritance of an X-linked recessive disease in two male patients and two female obligate carriers. There are two possible ways the c.78+1G>C splice site mutation may lead to frameshift and introduce a premature termination codon at the beginning of exon 3: after activation of the next cryptic splice site by a 10 bp insertion or after exon skipping by a 26 bp deletion. The splice site mutation in the second intron of *RS1* identified in these XLRS patients is practically identical to the N-ethyl-N-nitrosourea (ENU) induced splice site mutation in the mouse model of XLRS described by the Tennessee Mouse Genome Consortium. The genetic findings of the mutant mouse model confirm and support our human results.

## Introduction

X-linked juvenile retinoschisis (XLRS; OMIM 312700) is the leading cause of juvenile macular degeneration and is limited almost exclusively to males. It is caused by mutations in the *RS1* gene in Xp22.2 [1]. Affected males show radially oriented intraretinal foveomacular cysts (spoke-wheel pattern) in different retinal layers from inner to outer layers in the earlier stage. In later stages of the disease the coalescing cysts form a large central cavity, which can ultimately progress into nonspecific macular atrophy [2-5]. Approximately half of cases have bilateral peripheral retinoschisis in the inferotemporal part of the retina [6]. Boys are usually diagnosed with uncorrectable visual impairment between the ages of 5 and 10 years [6]. Disease severity and progression are highly variable even within families, and complications such as vitreous hemorrhage, choroidal sclerosis, retinal detachment, and neovascular glaucoma may occur [6-8]. Full-field electroretinograms (ERGs) of the affected individuals are characterized by a reduction in the amplitude of standard combined b-wave and a relative preservation of the a-wave, which is called electronegative ERG (b/a wave ratio≤1) [9,10]. The a-wave can also be reduced with age due to increasing RPE atrophy or photoreceptor involvement [6,10]. The multifocal ERGs (mfERG) of patients are characterized by diminished response densities, mainly in the central rings with delayed implicit times [9]. Thus ERG is a useful method to make the diagnoses of XLRS, but it is not a specific indicator of XLRS [9].

**Figure 1:**
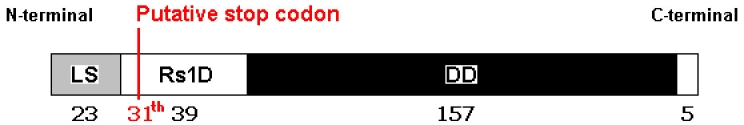
Schematic diagram of RS1 protein. The following abbreviations were used: LS represents leader sequence (23 amino acids (AAs)), Rs1D represents Rs1 domain (39 AAs), DD represents discoidin domain (157 AAs), and there is a five AA segment present on the C-terminal side of the DD. The red line marks the position of the putative premature stop codon at the beginning of exon 3.

The *RS1* gene that causes XLRS (localization: Xp22.2, GenBank AF014459) was identified in 1997 by positional cloning [1]. Since then numerous inactivating mutations have since been found and they are summarized in the retinoschisis sequence variation database. *RS1* has six exons and encodes a 224 amino acid (AA) secretable extracellular adhesion protein, called retinoschisin (RS1). RS1 is primarily present in photoreceptors and bipolar cells and it interacts with the surface of these cells to stabilize the organization of the retina [11]. During the early stages of retinal development, ganglion cells also express RS1. Müller cells neither express RS1 nor are involved in its transport [12]. RS1 may be involved in cellular adhesion and cell-cell interactions on membrane surfaces [8,13]. The predicted RS1 protein contains three domains: a 23 amino acid (AA) secretory leader sequence (LS, encoded by exon 1–2), a 39 AA Rs1 domain (Rs1D, encoded by exon 3) responsible for oligomerization, and a highly conserved 157 AA discoidin domain (DD, encoded by exon 4–6; Figure 1) [10,13-16]. There is a 5 AA segment at the C-terminal end of the protein. The highly conserved, hydrophobic N-terminal 23-residue secretory leader signal sequence mediates the protein export [9,13,14]. It is predicted to be cleaved by a signal peptidase as part of the protein secretion process [14]. The mature protein has a calculated size of 201 AA and contains the main structural feature of RS1, the highly conserved DD, which is shared with several other proteins [9]. The dysfunctional, defective RS1 protein is probably accumulated both extracellularly and intracellularly, eventually leading to cystic-like spaces and schisis formation in several different layers of the retina [7,17].

Most of the mutations are missense mutations, and most disease-causing mutations are localized to exon 4–6 of *RS1* within the DD. Exons 1–3 tend to have mainly translation-truncating nonsense mutations [18,19]. In addition to these mutations, insertions, deletions, duplications, intragenic deletions, splice site and frameshift mutations have also been identified in XLRS patients [1,2,18,19].

The phenotype (as measured by optical coherence tomography (OCT), fundoscopy, and ERG) shows a wide interocular and intrafamilial variability even in the case of the same mutation [2,20]. Thus far no real genotype-phenotype correlation has been detected [20,21].

Here we demonstrate the phenotype and genotype of a novel putative splice donor site mutation in a Hungarian family and compare their genetic background with a similar ENU-induced mutation in mouse, generated by the Tennessee Mouse Genome Consortium [22].

## Methods

### Clinical studies

All studies were conducted in accordance with the tenets of the Declaration of Helsinki. Voluntary informed consent was obtained from all participating individuals (or from their parents) after providing a full explanation of the procedures. The molecular genetic examinations were approved by the Ethics Committee for Human Genome Research of Semmelweis University. We analyzed this family only, because no other family was found with this splice site mutation. There were no more relatives in the family. In spite of the low pedigree size, founding the mutation in the *RS1* gene of the X-chromosome can prove the X-linked inheritance in this family. Our pedigree excludes the dominant inharitance pattern and on the basis of our knowledge there was no other inheritance pattern published if an *RS1* mutation is found. The rare cases, like consangious marriage, X-chromosome inactivation, Turner syndrome can be excluded. So the inheritance can be X-linked recessive only.

**Figure 2:**
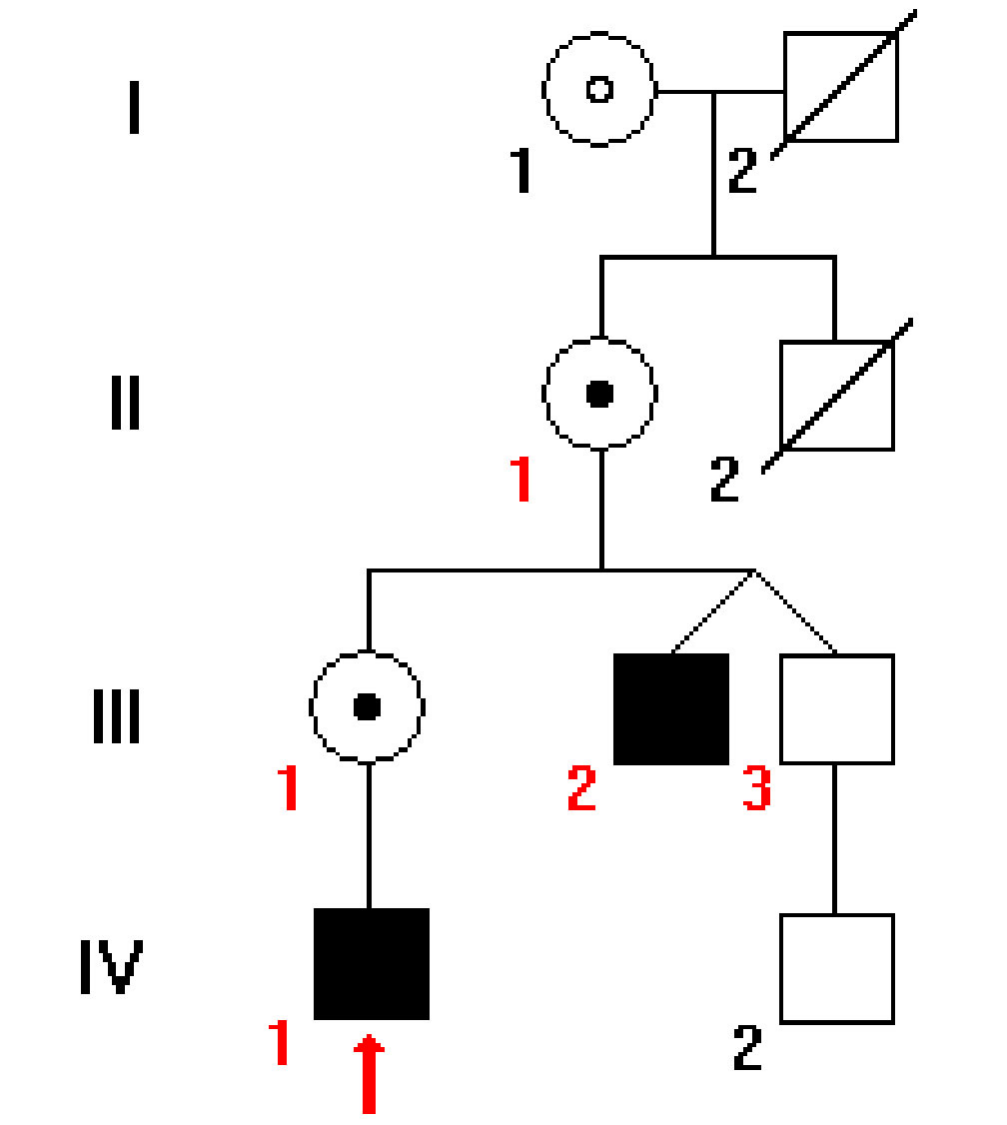
Pedigree of the family with X-linked juvenile retinoschisis. Black boxes represent affected males, while circles with a black dot in the center represent carrier females by sequence analysis. The circle with an open dot represents a female that is expected to be an obligate carrier female. Red numbers mark family members who underwent complete clinical and molecular genetic examinations. Red arrow points to the proband. Slashed boxes are deceased family members. Patients III/2 and III/3 are fraternal twins.

A Hungarian family (Figure 2) with XLRS was referred to the study by the Department of Ophthalmology, Semmelweis University, Budapest, Hungary. Complete ophthalmic examinations including OCT, full-field and multifocal ERGs were performed on three males and two female obligate carriers. Two of the three males (III/2 and III/3) are fraternal twins and only one of them had visual difficulties.

Best corrected visual acuity (BCVA) was determined using a Snellen chart. Dilated indirect ophthalmoscopy of the posterior pole was performed with fundus photography.

OCT was performed in both eyes of each family member, using six 6-mm long OCT scans centered on the fovea by a Stratus OCT device (Carl Zeiss Medical Inc., Dublin, CA). The foveal thickness was calculated on the basis of OCT scans by the built-in software of the device and shows the retinal thickness between the internal limiting membrane and the pigmentepithel layer at the fovea. The FT map is a color-coded map of foveal thickness and uses to evaluate the size of macular edema, but also suitable to visualize macular cyst and check the central fixation. The total macular volume was also calculated by the built-in software and shows the volume between the above mentioned two layers at the macula and also capable to evaluate the size of edema or cyst.

Full-field ERGs (Roland Consult RetiPort, Wiesbaden, Germany) with white single flash stimuli were recorded according to International Society for Clinical Electrophysiology of Vision standards [23,24]. Pupils were dilated by instilling one drop of cyclopentolate 0.5% solution in each eye, and corneal jet electrodes were placed after instilling 0.4% oxybuprocaine chlorate as a topical corneal anesthesia. The amplitudes and implicit times of scotopic bright-flash (maximal response) a- and b-waves were measured, and the ratios of the b/a amplitudes were calculated.

The multifocal ERGs (Roland Consult RetiScan, Wiesbaden, Germany) were recorded with the same electrodes, using a 21” video stimulating display. Before insertion of the contact lens electrode, the subjects were optically corrected for the viewing distance if it was possible. The central 60° diameter part of the retina was stimulated in each eye separately by 61 hexagons under photopic conditions. Amplitudes and implicit times of the b-waves (P1) of first order kernels were measured.

All OCT and ERG results were compared with the values of 35 male and 35 female control subjects, with a mean age±SD: 22±10 years, respectively. To confirm the pathogenetic effects of the identified mutation 50 control male subjects (including the previous mentioned 35 control males), with a mean age±SD: 22.8±10.3 years were genetically examined. All controls were examined using the same clinical protocol. All of them had normal vision, had not any acquired or genetically inherited eye diseases and not the members of XLRS families. Inclusion criteria in the normative database were: preserved vision and absence of any acquired or genetically inherited eye diseases. A simple comparison was performed with the mean±2SD values of the normative database.

### Molecular genetic studies

Peripheral blood (6 ml) was collected in EDTA-K3 tubes from all family members and 50 healthy male controls. Genomic DNA was isolated from leukocytes according to the manufacturer’s instructions, using the QIAamp DNA Mini Kit (Qiagen GmbH; Hilden, Germany). Isolated DNA samples were stored at a temperature -20°C. The 50 male controls include the 35 ones from the OCT and ERG controls. For mutation screening all exons and the flanking intronic regions of *RS1* were amplified by the polymerase chain reaction (PCR) process using standard primers. Details of the primers, size of the amplified fragments, and annealing temperatures are given in Table 1.

**Table 1:** Sequences of primers used to amplify the coding regions of the *RS1* gene.

**Exon**	**Primers (5′-3′)**	**Product size (bp)**	**Ta (°C)**
1	F: CTCAGCCAAAGACCTAAGAAC	216	58
	R: GTATGCAATGAATGTCAATGG		
2	F: GTGATGCTGTTGGATTTCTC	176	56
	R: CAAAGTGATAGTCCTCTATG		
3	F: CGATGCATAAGGACTGAGTGTGATC	377	50
	R: GCATTAACATAGGCTTAC TAATAG		
4	F: CGTGAGTAGTGAACCGTTGAAGAC	381	50
	R: ACGCTGGTAGAGAGGCCTAT		
5	F: GCAAGTTAAGTATAACGGAAGCTG G	508	50
	R: GGAAAGCGCAGATGATCCACTGTG		
6	F: GCAAACTGCTTTAACTAC TTCC	427	50
	R: CCAGCACTGCAGTTACAATTGC		

Shown are primer sequences, annealing temperatures, and anticipated size of the amplified product for the different fragments of *RS1* studied. The following abbreviations were used: base pair (bp) and annealing temperature (Ta). The template sequence is available at NCBI, GenBank accession number: RS1: NM_000330.

PCR was performed using genomic DNA in a Termo Hybaid PxE thermal cycler (Thermo Hybaid, Franklin, MA). The reaction volume of 50 µl contained the following: 5X GoTaq Reaction Buffer (pH 8.5, containing 1.5 mM MgCl_2_; Promega Corporation, Madison WI), 25 pmoles of each primer (Invitrogen Life Technologies, Glasgow, UK), 0.2 mM each dNTP (Promega), and 2.5 U GoTaq Polymerase (Promega). PCR cycles for the retinoschisin gene were as follows: pre-denaturation at 95 °C for 2 min, followed by 35 cycles of denaturation at 95 °C for 1 min, annealing at the appropriate temperature for 1 min, and 1 min elongation at 72 °C. The final extension was performed at 72 °C for 10 min.

The amplicons were analyzed on 1% agarose gel and stained with ethidium bromide. The resulting DNAs were purified using Roche High Pure PCR Purification Kit (Roche Diagnostics GmbH, Mannheim Germany) according to the manufacturer’s protocol.

PCR-amplified DNA of *RS1* were sequenced by direct nucleotide sequencing using the Big Dye Terminator Cycle-Sequencing v3.1 Kit (Applied Biosystems, Foster City, CA) and run on an automated sequencer (ABI Prism® 310 Genetic Analyzer; Perkin Elmer™; Applied Biosystems).

The results were compared with the reference sequence from the X-linked retinoschisis sequence variation database and the sequence of the 50 controls.

The putative donor splice site was predicted with NNSPLICE, 0.9 version, at the website of the Berkeley Drosophila genome project.

## Results

### Clinical studies

The clinical data of patients are reported in Table 2. A pedigree of the family members involved in the study is given in Figure 2.

**Table 2:** Clinical data and molecular genetic examination results of the family with XLRS and our control group.

**Patients/** **carriers number** **(Eye)**	**Mutation**	**Gender**	**Age (yrs)**	**Age of onset (yrs)**	**Macular abnormalities**	**BCVA**	**Spherical corrections (D)**	**OCT**		**Full-field ERG**		**Scot. max. b/a ratio**	**Multifocal ERG**	
								**FTmax (μm)**	**TMV (mm3)**	**Scot. max. a-wave ampl. (μV)**	**Scot. max. b-wave ampl. (μV)**		**Summed ampl. (μV)**	**Summed impl. (ms)**
IV/1 (OD)	c.78+1G>C	male	7	7	foveal schisis	0.25	1.75	505	8.5	253	339	1.34	49	37
(OS)				7	foveal schisis	0.3	1.75	591	9.05	253	360	1.43	42	37
III/1 (OD)	c.78+1G>C	female	31	-	-	1	-	152	6.51	352	815	2.32	140	36
(OS)				-	-	1	-	156	6.5	372	811	2.18	145	35
III/2 (OD)	c.78+1G>C	male	25	6	foveal schisis	0.42	-	339	6.97	336	340	1.01	46	40
(OS)				6	foveal schisis	0.42	-	621	8.41	302	304	1	42	42
III/3 (OD)	-	male	25	-	-	1	-	152	6.74	-	-	-	-	-
(OS)				-	-	1	-	171	6.97	-	-	-	-	-
II/1 (OD)	c.78+1G>C	female	50	-	-	1	-	189	6.67	238	515	2.16	99	37
(OS)				-	-	1	-	175	6.67	291	560	1.92	94	37
Control (n=70)	-	50-50%	22±10	-	-	0.98±0.06	-	157±17	6.96±0.26	314±65	593±86	1.91±0.35	118±25	33±1

No patients displayed peripheral schisis. Patient IV/1 had the additional symptom of Mizuo-Nakamura phenomenon. Control values are means ± standard deviations (SD). YRS indicate years, BCVA represents best corrected visual acuity, D represents diopter, OCT represents optical coherence tomography, FTmax represents maximal foveal thickness, TMV represents total macular volume, and ERG represents electroretinography. The significantly increased FT and TMV values of OCT (p<0.05) and the significantly (p<0.05) decreased BCVA values, full-field and multifocal ERG amplitudes demonstrate well the morphological and functional impairment of the retina in case patients with XLRS.

**Figure 3:**
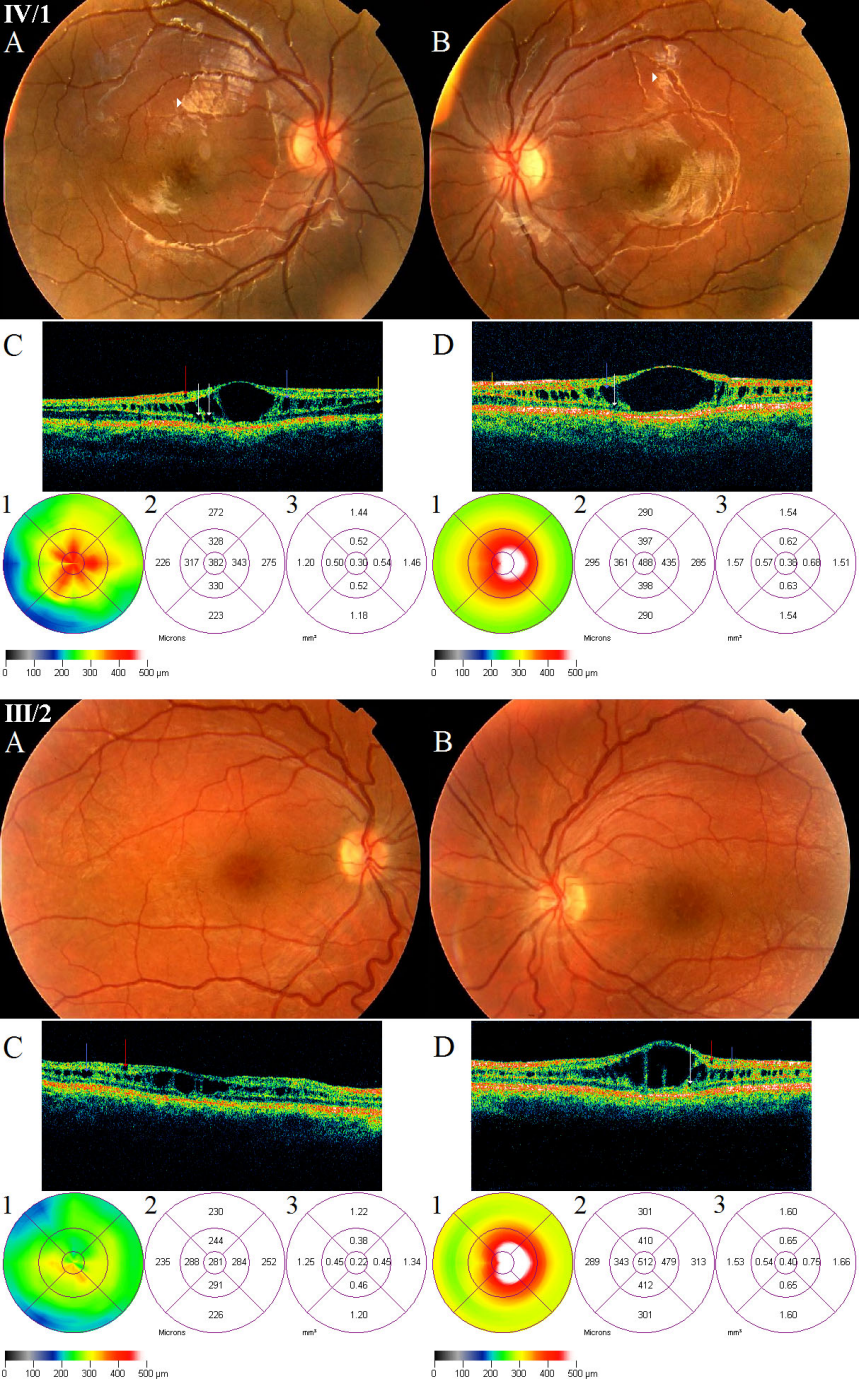
Fundus photographs and optical coherence tomography images of patients IV/1 and III/2 suffering in X-linked juvenile retinoschisis. In patient IV/1, the fundi show radially oriented intraretinal foveomacular cysts in a spoke-wheel configuration, with the absence of foveal reflex (IV/1 **A, B**). A golden-yellow reflex called Mizou-Nakamura phenomenon is seen on the posterior pole of both eyes of patient IV/1 marked by white arrowheads (**A, B**). The OCT images of him (IV/1 **C, D**) reveal retinoschisis in the inner nuclear layer (marked by blue arrow), in the photoreceptor layer (marked by yellow arrow) and some cysts in the outer plexiform layer (marked by white arrows) in both eyes, and one cyst in the ganglion cell layer (marked by red arrow) in the right eye. His OCT scans and foveal thickness maps show significant diffuse thickening of the right fovea (IV/1 **C**, C1-2), and because of the huge central cyst the significant pronounced thickening of the left fovea (IV/1 **D**, D1-2) compared with the controls. The eccentric fixation is clearly identifiable on his left FT map (IV/1 D1). In patient III/2, the fundi show spoke-wheel configurations in the foveas with the absence of foveal reflex (III/2 **A, B**). The OCT images reveal retinoschisis in the inner nuclear layer (pronounced in the left eye), small cysts in the ganglion cell layer of both eyes (III/2 **C, D**) and one cyst in the outer plexiform layer of left eye (III/2 D). His OCT scans and foveal thickness maps show significant diffuse thickening of the right fovea (III/2 **C**, C1-2), and because of the huge central cyst the significant pronounced thickening of the left fovea (III/2 **D**, D1-2). The eccentric fixation is clearly identifiable on his left FT map (IIII/2 D1).

Fundoscopy showed typical bilateral spokewheel-like foveal retinoschisis (Figure 3) characterized by radially arranged microcysts in the inner layers of the retina with the absence of foveal reflex in patients IV/1 and III/2. Neither peripheral retinoschisis nor any retinal tears or retinal detachments were evident. Golden-like fundus reflex, also called the Mizuo-Nakamura phenomenon, was found in both fundi of patient IV/1.

BCVA parameters of male patients, which varied from 0.25 to 0.42, were below the normal BCVA range of 0.98±0.06. At his first examination, patient IV/1 could hardly find the numbers on the Snellen chart. His BCVA was 0.25 in the right eye and 0.3 with eccentric fixation in the left eye. One year later, the fundoscopic findings had not changed, but his visual acuity had improved: BCVA in this patient’s right eye and left eye were both 0.42 without any correction and with eccentric fixation in the left eye.

OCT images of patient IV/1 showed huge low reflective cysts in the fovea surrounded perifoveally by many smaller ones in the inner nuclear layer (INL), and another deeper schisis in the photoreceptor layer (Figure 3). Next to the huge central cysts, some cavities were evident in the outer plexiform layer (OPL). The cysts were separated by multiple, highly reflective, vertical, thin-walled strands. In the right eye there was a small cyst in the ganglion cell layer also. Both OCT scans and the foveal thickness map proved the eccentric fixation in the left eye.

OCT images of patient III/2 (Figure 3) showed a flattened schisis with smaller cysts bordered by vertical bridging retinal elements in the INL in the right eye. In the left eye there were huge centrally localized cysts besides the smaller ones in the INL bordered by vertical bridging retinal strands with eccentric fixation in the left eye. Small cysts could also be found in the ganglion cell layer in both eyes. Foveal thickness and total macular volume parameters of the two patients were increased (FT: 328% of the normative values; TMV: 118%). OCT parameters of other family members were normal.

Full-field ERG recordings of patient IV/1 and III/2 (Table 2) showed markedly reduced standard combined b-wave amplitudes (57% of the normative values) with the relative preservation of standard combined a-wave amplitudes (91%) in both eyes. The standard combined b/a amplitude ratios were also decreased (63%), but the typical “negative-type” ERG was not observed.

**Figure 4:**
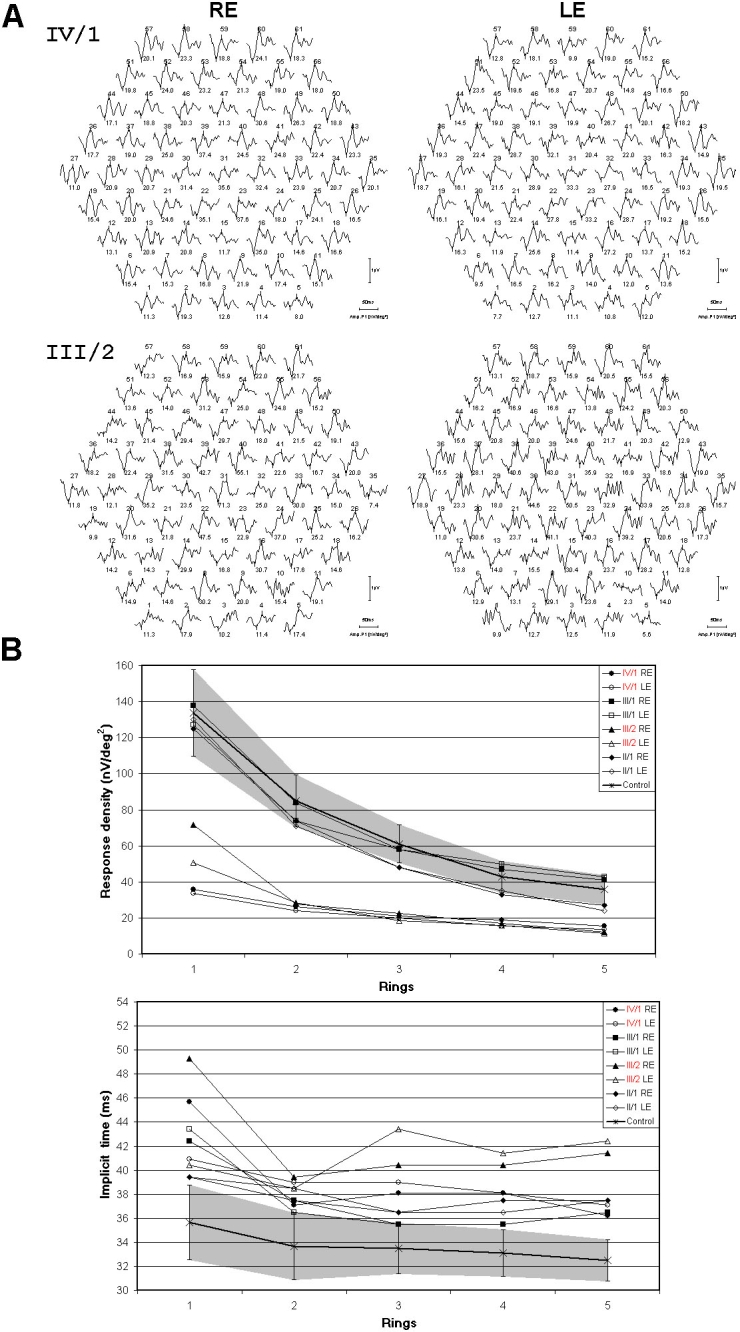
Multifocal electroretinography results of the two patients, carriers, and controls. **A:** Trace arrays of patients IV/1 and III/2 with 61 elements. **B:** Response densities (RDs) and implicit times of multifocal electroretinographs for five eccentric rings in patients (marked red), carriers, and controls. Gray area represents the 95% confidence interval of our control database, the single control line in the center of the gray area represents the average of the set of control patients, while error bars represents ± standard deviation (SD). Response densities (RDs) of patients were decreased in all rings, while carriers’ RDs were within the normal range. Implicit times of patients and carriers were delayed in all rings. The following abbreviations were used: right eye (RE) and left eye (LE).

Response densities (RDs) of mfERGs of patient IV/1 and III/2 (Figure 4A,B) were characterized by decreased P1 (b) amplitudes (IV/1>III/2 in the first rings) across the whole examined retinal area, but mainly in the three central rings as compared with controls. RDs of carriers were within the normal range. Implicit times of patients and carriers were delayed in all rings compared with controls.

### Molecular genetic studies

**Figure 5:**
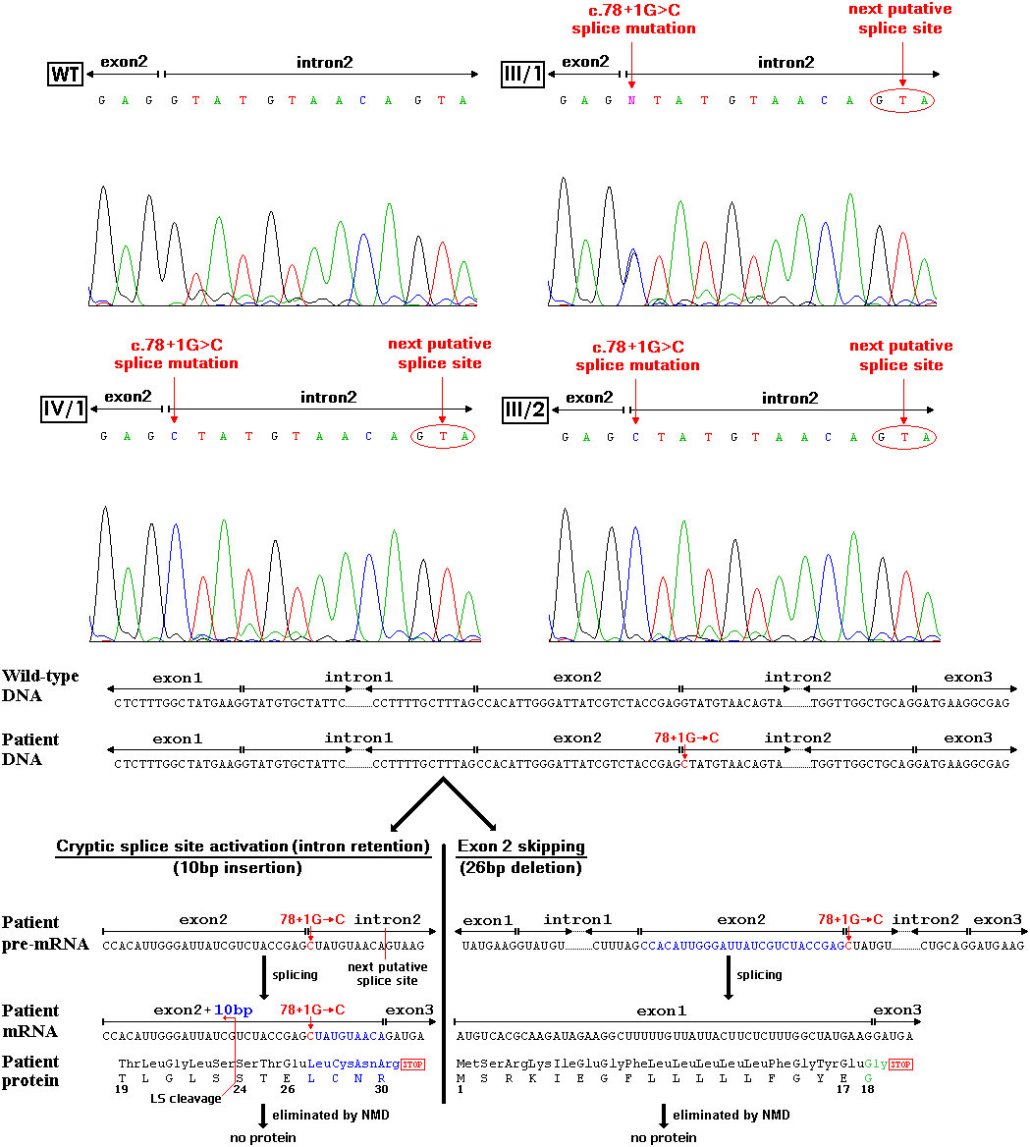
DNA sequence in wild type (WT), in III/1 heterozygote carrier mother, in IV/1 and III/2 hemizygote patients. In case of patients with c.78+1G→C mutation there may be two possible ways during RNA splicing. The first possible way is the partial intron retention by the activation of a cryptic splice site. Mutating the conserved splice donor site from GT to CT, a novel cryptic site may become activated during RNA splicing, and thus the splice is probably shifted by ten nucleotides (CTA TGT AAC A) downstream to the next GT in the intronic sequence. Thus exon 2 gets longer by 10 nucleotides (marked blue in the picture), which results in a frameshift and finally cause a premature termination codon (PTC) at the beginning of exons 3 (after codon 30). In this way the truncated RS1 protein would contain only 30 AAs, but previously the truncated mRNA may probably be eliminated by the nonsense-mediated mRNA decay (NMD). The second possible way is the exon 2 skipping with a 26 bp deletion (marked blue in the picture) at position 52. It may result in a frameshift and a new PTC at the beginning of exon 3 at the same position like in case the above described partial intron retention. In this way the truncated RS1 protein would consist of only 17 AAs and an additional new one (marked green in the picture) at the C terminal. But previously, like in the first hypothetical way, the truncated mRNA may probably be eliminated by the nonsense-mediated mRNA decay resulting in no functional RS1 protein.

A novel putative *RS1* splice donor site mutation (c.78+1G>C) of intron 2 was identified in the two male patients (III/2, IV/1) and in two female carriers (III/1, II/1; Figure 2 and Figure 5; Table 1). There was no other disease-causing mutation in the exons or in other flanking introns of *RS1*. The asymptomatic fraternal twin (III/3) had no mutation in *RS1.* There were no any SNPs found in the disease family in or near the *RS1* gene locus.

## Discussion

The symptoms, the morphological (OCT) and functional (ERGs) phenotypes of patients IV/1 and III/2 corresponded with XLRS. These patients had characteristic bilateral spokewheel-like pattern of the macula without any peripheral retinoschisis.

The rare golden-like fundus reflex on the fundi of patient IV/1 (Mizuo-Nakamura phenomenon) was previously described in the dark-adapted retina immediately or shortly after exposure to light. The golden-like fundus reflex is primarily observed in Oguchi's disease and in rare cases in XLRS [25]. This reflex is probably attributed to a potassium imbalance in the inner retina as a result of a decreased potassium scavenging capacity of retinal Müller cells [25].

Two affected patients had decreased visual acuity bilaterally. Patient IV/1 showed an improvement of visual acuity in both eyes at a one-year follow-up. It may presumably be due to the more efficient use of the ectopic fovea. It can occur only in young age as a result of the plasticity of the developing visual system [4,26].

OCT images of the two patients showed huge low reflective cysts in the fovea surrounded by many smaller ones in the INL perifoveally, and another deeper schisis in the photoreceptor layer, where normally the retinoschisin protein can be detected in the highest quantity. FT and TMV of the two patients were increased, and the eccentric fixation was easily detected on the OCT macular thickness map (Figure 3). Using the retinoschisis classification system developed by Prenner at al. [27], we classified each eye of patients IV/1 and III/2 as type 3.

The standard combined responses of full-field ERGs of the two patients had a reduction in b-wave amplitude and a relative preservation of the a-wave, leading to a reduction in the b/a ratio (negative-type ERG). The standard combined b/a amplitude ratios were lower in the older patient. The negative-type ERG was described as characteristic of XLRS; however it is not specific, as it can be found in other retinal diseases (e.g., congenital stationary night blindness, Oguchi disease, fundus albipunctatus, Batten disease, central retinal artery occlusion, cancer-associated retinopathy, melanoma associated retinopathy, Duchenne muscular dystrophy) [28].

In the mfERGs, first order kernels of the two patients showed decreased P1 (b) amplitudes in all rings, but mainly in the three central rings. Implicit times were delayed in all rings.

During the aging of patients with XLRS, the size of foveal cysts and the ERG b-wave amplitudes decrease [5,10,29,30]. Comparing with patient IV/1, the flattened cysts, lower OCT values, and decreased standard combined b/a amplitude ratios of patient III/2 were probably due to the age effect [5,10,29,30].

The cell origin of full-field and mfERG responses is concluded as the same [31]. The a-wave originates from photoreceptors, while the b-wave from ON- and OFF-bipolar cells with a smaller contribution of cone photoreceptors [31]. Accordingly the decreased standard combined b-waves and the decreased P1-waves of our patients suggest that the retinal damage affects mainly the INL with bipolar cells and less the photoreceptor layer. This corresponds with our OCT findings as more pronounced schisis was found in the INL and smaller ones in the photoreceptor layer. The highest quantity of RS1 protein was also detected in these two layers by immunohistochemistry [11,32].

As a new finding we described a novel putative splice mutation with a G to C transversion at the conserved GT splice donor site of intron 2 leading to 5′ splicing mutant variants of retinoschisin. This may happen by cryptic splice site activation or by exon skipping (Figure 5) described below:

### Cryptic splice site activation (intron retention)

Conserved GT signals are important for normal gene expression as they are involved in mRNA processing [33]. By mutating a conserved splice donor site from GT to CT, a novel cryptic site may become activated during RNA splicing, and thus the splice is probably shifted by ten nucleotides (donor site prediction score: 0.91) downstream to the next GT in the intronic sequence. Consequently, exon 2 becomes longer by ten nucleotides (**C**UAUGUAACA) inserted between positions 78 and 79, and the insertion encodes Leu+Cys+Asn amino acids and a residual adenine (A) nucleotide. The frameshift hypothetically results in a consecutive premature termination codon (PTC) in the beginning of exon 3 (after codon 30). The mRNA with its PTC is probably destroyed by a quality-control surveillance mechanism called nonsense-mediated mRNA decay (NMD). It protects the cell from mutations that could yield to truncated proteins by eliminating mRNA that contains a PTC [34].

### Exon skipping

Nonsense, but not missense mutations, can alter splicing pathways, resulting in failure to include the affected exon in the final mRNA (a phenomenon known as exon skipping). The presumed 26 bp deletion at position 52 (exon 2 skipping) may cause frameshift and a PTC at the beginning of exon 3, as with the case of the 10 bp insertion described in the previous section. The mRNA with PTC is probably eliminated by NMD.

PTCs that occur because of the frameshift caused either by the 10 bp insertion or the 26 bp deletion may result in the total absence of RS1 protein by NMD and the resultant development of XLRS.

In a comparison of the clinical results of the two patients with our own retinoschisis database, we found the absence of the entire RS1 protein (completely lacking DD) did not seem to be accompanied by more severe symptoms than the less serious structural lesions of RS1 protein caused by other mutations. This is in agreement with previous findings for many X-linked recessive diseases: Considering that female carriers are unaffected, it seems that the absence of a functional RS1 protein in males, rather than the presence of a gain-of-function mutant protein, is responsible for retinoschisis in affected males [35]. Consequently, the main point in avoiding retinoschisis is the presence of the functional RS1 protein.

The Tennessee Mouse Genome Consortium generated the *44TNJ* mutant mouse, the first murine model of X-linked retinoschisis in which the gene is expressed by using an ENU-based mutagenesis screen to produce recessive mutations [22]. The retinoschisis-1 homolog (*Rs1h*) cDNA, which was reverse transcribed from mRNA isolated from eyes, and the genomic DNA, obtained from tails, were sequenced directly. The gene *Rs1h* revealed a T->C splice donor site mutation at the second base of intron 2. This generated splice site mutation is very similar to our human mutation. The amplification of the mutant *Rs1h* cDNA by PCR revealed two new splice products in the retina in addition to the wild type. The first alternative transcript had a 10 bp insertion between positions 88 and 89, leading to elongation of exon 2 by the first 10 nucleotides of intron 2. The second alternative transcript had a 26 bp deletion at position 62 (entire exon 2). Both splice variants lead to frameshift and PTC at the beginning of exon 3. In both cases another open reading frame was found after only a few bases.

On the basis of the simultaneous presence of two different types of mRNA in 44TNJ mutant mouse and the very similar mutations, we hypothesized that there may also be two different types of mRNA simultaneously in our XLRS patients.

Since identification of RS1 splice variants in human is difficult because of the lack of tissue-specific cDNA sequence data, we feel further animal studies are needed to clarify whether the truncated Rs1h peptides or the peptides encoded by the new open reading frames are present in the retina or whether the mutant mRNAs are degraded by NMD. However, our findings are very similar to those found in the above mentioned animal model of XLRS and may help to understand the genetic background of the disease in our family with XLRS.
